# Dynamic Vascular Imaging Unveils Popliteal Artery Entrapment Syndrome in an Atypical Patient: A Case Report

**DOI:** 10.1155/cris/9019853

**Published:** 2026-09-08

**Authors:** Elie Alam, Hadi Saada, Joya Ghaleb, Rita-Nour Awad, Nadia Katrib, Sandra Rizk, Moro Chehine, Ghassan Nabbout, Zaki Samia

**Affiliations:** ^1^ Department of Orthopedic Surgery, Faculty of Medicine and Medical Sciences, University of Balamand, Koura, North Governorate, Lebanon, balamand.edu.lb; ^2^ Department of Biomedical Sciences, Faculty of Medicine and Medical Sciences, University of Balamand, Koura, North Governorate, Lebanon, balamand.edu.lb; ^3^ Department of Vascular Surgery, Nini Hospital, Tripoli, North Governorate, Lebanon, hopitalnini.com

**Keywords:** case report, claudication, dynamic Doppler ultrasound, gastrocnemius hypertrophy, popliteal artery entrapment syndrome, surgical decompression, vascular compression

## Abstract

Popliteal artery entrapment syndrome (PAES) is a rare vascular disorder resulting from abnormal anatomical relationships between the popliteal artery and adjacent musculotendinous structures. This case highlights a 39‐year‐old female with a 10‐year history of exertional calf pain, worsened by walking and stair descent. Multiple static imaging tests failed to diagnose her condition. However, dynamic Doppler ultrasound revealed arterial occlusion during dorsiflexion of the ankle, and dynamic arteriography confirmed PAES. Surgery uncovered a fibrous band and hypertrophied medial gastrocnemius muscle compressing the artery. She underwent fibrous band release, muscle resection, and arterial decompression, resulting in full symptom resolution and restored blood flow. This case underscores the diagnostic challenges of PAES in atypical patients, where lack of vascular risk factors and normal resting studies can mislead clinicians. Dynamic vascular imaging is vital for accurate diagnosis. PAES should be considered in all patients with unexplained exertional leg pain, and timely surgical treatment can yield excellent outcomes.

## 1. Introduction

Popliteal artery entrapment syndrome (PAES) is a rare vascular disorder resulting from abnormal anatomical relationships between the popliteal artery and adjacent musculotendinous structures [1]. Though once thought to mainly affect young male athletes, better awareness and imaging have demonstrated cases in broader populations. PAES arises from developmental anomalies involving the popliteal artery and the medial head of the gastrocnemius muscle (MHG) [2]. The Whelan classification defines six types of PAES based on the specific patterns of arterial entrapment (Table 1) [3].

**Table 1:** Whelan classification of PAES.

Type	Anatomical relationship
I	Abnormal medial course of popliteal artery around a normally positioned (MHG)
II	Abnormal lateral attachment of MHG on femur causing a medial and inferior popliteal artery path
III	Abnormal fibrous band or accessory muscle arising from the medial or lateral condyle encircling the popliteal artery that is normally positioned
IV	Deep or axial positioning of the popliteal artery within the fossa, entrapping it by popliteus muscle
V	Entrapped popliteal vein with the popliteal artery in any of the above scenarios
VI	Compression of both the popliteal artery and vein due to muscular hypertrophy

Types I–V of PAES are congenital, while type VI is acquired, often due to muscle hypertrophy in physically active individuals [4]. Symptoms typically arise when the entrapped popliteal artery becomes compromised during exercise or certain limb positions [2]. PAES affects about 0.6%–3.5% of the population, predominantly young, active males (~83%) [5]. The most common anatomical subtype remains debated, with types III and IV most frequently cited [3, 5, 6]. Although rare, PAES should be considered in young patients with unexplained leg claudication as early diagnosis and treatment are essential to prevent lasting vascular damage.

We report the case of a 39‐year‐old female diagnosed with PAES, highlighting an atypical demographic presentation of this condition.

## 2. Case Presentation

A 39‐year‐old previously healthy woman presented with a 10‐year history of exertional left calf pain and heaviness, triggered by walking long distances or descending stairs and relieved by rest. Despite undergoing multiple imaging studies, including Doppler ultrasonography and MRI, no definitive diagnosis had been made. Over time, the symptoms worsened, limiting her daily physical activities.

On physical examination, the patient described a cramping pain localized to the left calf that was reproducibly exacerbated by active dorsiflexion of the ankle. Baseline vascular assessment at rest, including pulse examination and resting Doppler evaluation, was unremarkable, with preserved distal perfusion and no evidence of fixed arterial obstruction.

**Figure 1:**
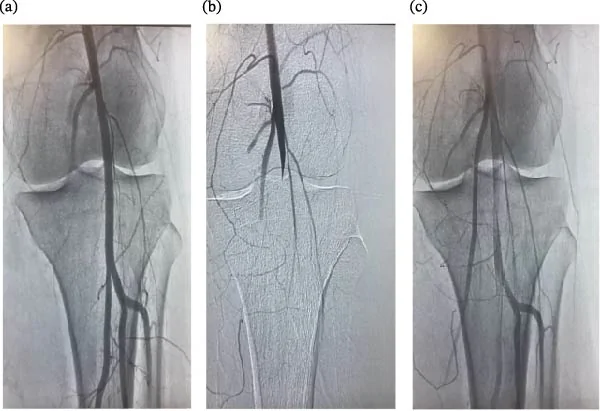
Dynamic CT angiography of the left lower limb. (a) Normal popliteal artery flow during plantar flexion. (b) Arterial occlusion during foot dorsiflexion. (c) Restoration of blood flow after release of dorsiflexion.

Given the dynamic nature of the symptoms, further provocative vascular imaging was performed. Dynamic Doppler ultrasonography demonstrated normal popliteal artery flow at rest; however, complete cessation of arterial flow was observed during active dorsiflexion of the ankle, strongly suggesting positional vascular compression. To further characterize the lesion and confirm the diagnosis, digital subtraction angiography (DSA) was subsequently obtained. During digital subtraction arteriography, dynamic provocative maneuvers were performed by asking the patient to actively dorsiflex the ankle while sequential angiographic images were acquired. Imaging was obtained both in the neutral resting position and during forced dorsiflexion, which reproduced the vascular compression and demonstrated a complete transient occlusion of the popliteal artery. Restoration of normal arterial flow was documented after relaxation and plantarflexion of the ankle (Figure 1a–c). These dynamic imaging findings were highly consistent with PAES.

The contralateral lower limb was clinically asymptomatic. Dynamic vascular assessment of the right leg demonstrated normal arterial flow both at rest and during provocative maneuvers, with no evidence of vascular compression or entrapment.

Surgical exploration through a posterior S‐shaped incision revealed a thick fibrous band compressing the popliteal artery and a hypertrophied MHG.

**Figure 2:**
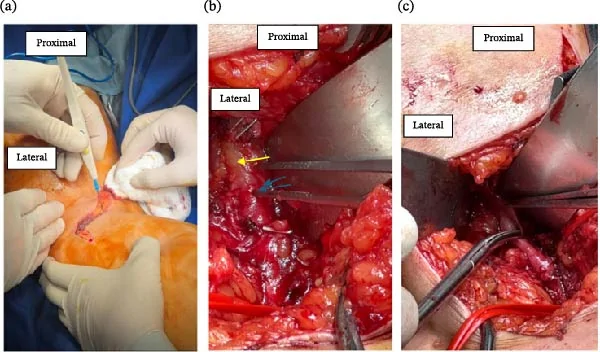
Intraoperative findings and surgical release of the popliteal artery. (a) “S” incision for popliteal fossa exploration. (b) Identification of a thick fibrous band (blue arrow) compressing the popliteal artery (yellow arrow) (c) Release of the fibrous band.

**Figure 3:**
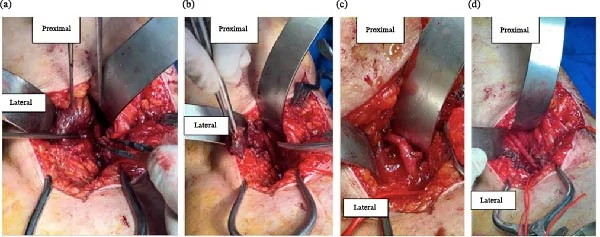
Surgical findings and intervention for popliteal artery compression. (a) Hypertrophied medial head of the gastrocnemius muscle compressing the popliteal artery. (b) Partial excision of the muscle. (c) Ligation and release of collateral branches of the popliteal artery. (d) Restoration of unrestricted arterial movement.

**Figure 4:**
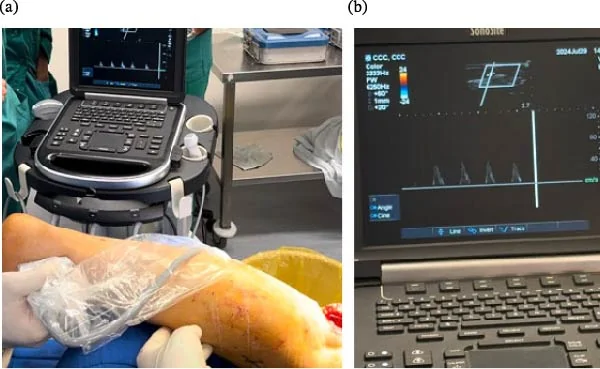
Intraoperative Doppler ultrasound after artery release. (a) Absence of arterial compression during foot dorsiflexion. (b) Restoration of blood flow wave.

Careful circumferential dissection of the popliteal artery was performed under direct visualization to avoid intimal injury or excessive traction. Vessel loops were used to gently mobilize and protect the artery throughout the decompression procedure. Both the fibrous band and part of the muscle were excised. Intraoperatively, preservation of collateral arterial branches was attempted whenever feasible; however, selective ligation of a limited number of branches was deemed necessary to achieve complete decompression and prevent recurrent positional compression. No major collateral vessels were sacrificed (Figures 2 and 3). Intraoperative Doppler confirmed restored blood flow without recurrence of compression during dorsiflexion (Figure 4a,b).

Postoperatively, the patient experienced a complete resolution of symptoms. Postoperative follow‐up was performed using Doppler ultrasound with dynamic provocative maneuvers, including active dorsiflexion of the ankle. The examination demonstrated sustained arterial patency without recurrent compression or flow limitation during positional testing.

The patient was advised to progressively resume physical activity as tolerated. No permanent activity restrictions were recommended.

## 3. Discussion

PAES is an uncommon but important cause of intermittent claudication, particularly in younger individuals without traditional cardiovascular risk factors. It results from external compression of the popliteal artery, most often by anomalous muscular or fibrous structures within the popliteal fossa [2]. The condition can be either congenital—arising from developmental abnormalities of the gastrocnemius or adjacent musculotendinous structures—or acquired through repetitive trauma or hypertrophy, particularly in athletes [4].

Most patients with PAES remain asymptomatic for years. However, symptom onset is often precipitated by exercise, which promotes hypertrophic changes in the gastrocnemius muscle that exacerbate vascular compression. In contrast to the classic profile of young male athletes, our case involved a middle‐aged, nonathletic female, thereby highlighting the importance of considering congenital PAES even in atypical populations.

There have been similar cases reported in nonathletic and middle‐aged patients [4, 7–9], which, along with our case, support the notion that the demographic for PAES should be expanded. All of these cases also support the fact that PAES can remain silent or undetected until subtle anatomical changes or an increased activity level in adulthood unmask its vascular consequences.

The differential diagnosis for exertional calf pain is broad and includes peripheral arterial disease (PAD), musculoskeletal injuries (e.g., tendinitis, muscle strain), neurogenic claudication, chronic exertional compartment syndrome, and popliteal cysts [1]. However, our patient lacked conventional risk factors for PAD (e.g., smoking, diabetes, and hypertension), and MRI had previously ruled out musculoskeletal or soft‐tissue abnormalities. The chronicity, reproducibility of symptoms during dorsiflexion, and absence of findings at rest were key to redirecting attention toward a dynamic vascular cause.

Although several imaging modalities are available—including Doppler ultrasound, computed tomography angiography (CTA), and magnetic resonance angiography (MRA)—their sensitivity varies significantly. Static imaging alone is frequently nondiagnostic. Notably, the sensitivity of MRA and CTA has been reported as 54.5% and 51.7%, respectively [4, 7–9], while the combination of Doppler ultrasonography and MRI with provocative maneuvers demonstrates a sensitivity as high as 96.2% [9, 10]. In our patient, the diagnosis was missed in 2020 when imaging was performed at rest. Because PAES is fundamentally a positional and dynamic condition, static MRI may fail to demonstrate transient arterial entrapment, particularly in the absence of advanced vascular imaging protocols or provocative maneuvers. The turning point occurred when repeat Doppler ultrasound was conducted with dorsiflexion of the foot, reproducing symptoms and revealing complete dynamic occlusion of the popliteal artery, with prompt restoration of flow upon plantarflexion.

Static imaging modalities such as CTA and MRA often yield false‐negative results when evaluating PAES, a condition characterized by dynamic vascular compression. Unlike these static techniques, dynamic vascular imaging provides real‐time visualization, confirming proper limb positioning and directly demonstrating arterial entrapment [4, 9–11].

Although most patients experience excellent outcomes following surgical decompression, recurrence has been documented in ~9.2% of cases, with 7.1% requiring revision surgery [2]. This case illustrates how PAES can evade diagnosis for years in the absence of high clinical suspicion and underscores the importance of dynamic vascular assessment in patients with unexplained exertional leg pain and highlights that PAES can occur even in nonathletic individuals. Early recognition and timely surgical intervention are key to preventing irreversible vascular complications.

## Author Contributions

Concept and design: Elie Alam, Zaki Samia, and Moro Chehine. Acquisition, analysis, interpretation of data: Elie Alam, Hadi Saada, Joya Ghaleb, Rita‐Nour Awad, Sandra Rizk, and Nadia Katrib. Drafting of the manuscript: Elie Alam, Zaki Samia, and Ghassan Nabbout. Critical review of the manuscript for important intellectual content: Elie Alam and Ghassan Nabbout.

## Funding

No funding was received for this manuscript.

## Disclosure

After using ChatGPT, the authors reviewed and edited the content as needed and took full responsibility for the content of the publication.

## Consent

The patient consented to publish the case details and images.

## Conflicts of Interest

The authors declare no conflicts of interest.

## Data Availability

The data that support the findings of this study are available from the corresponding author upon reasonable request.
